# Multicenter phase II trial of accelerated cisplatin and high-dose epirubicin followed by surgery or radiotherapy in patients with stage IIIa non-small-cell lung cancer with mediastinal lymph node involvement (N2-disease)

**DOI:** 10.1038/sj.bjc.6603289

**Published:** 2006-08-08

**Authors:** E C J Phernambucq, B Biesma, E F Smit, M A Paul, A vd Tol, F M Schramel, R J Bolhuis, P E Postmus

**Affiliations:** 1 Department of Pulmonary Diseases, VU Medical Center, PO Box 7057, 1007 MB Amsterdam, The Netherlands; 2 Department of Pulmonary Diseases, Jeroen Bosch Hospital, PO Box 90153, 5200 ME 's-Hertogenbosch, The Netherlands; 3 Department of Pulmonary Diseases, Martini Hospital, PO Box 30033, 9700 RM Groningen, The Netherlands; 4 Department of Surgery, VU Medical Center, PO Box 7057, 1007 MB Amsterdam, The Netherlands; 5 Department of Surgery, Martini Hospital, PO Box 30033, 9700 RM, Groningen, The Netherlands; 6 Department of Pulmonary Diseases, St Antonius Hospital, PO Box 2500, 2430 EM, Nieuwegein, The Netherlands; 7 Department of Surgery, Jeroen Bosch Hospital, PO Box 90153, 5200 ME 's-Hertogenbosch, The Netherlands

**Keywords:** induction chemotherapy, non-small-cell lung cancer, N2-disease

## Abstract

To assess the therapeutic activity of accelerated cisplatin and high-dose epirubicin with erythropoietin and G-CSF support as induction therapy for patients with stage IIIa-N2 non-small-cell lung cancer (NSCLC). Patients with stage IIIa-N2 NSCLC were enrolled in a phase II trial. They received cisplatin 60 mg m^−2^ and epirubicin 135 mg m^−2^ every 2 weeks for three courses combined with erythropoietin and G-CSF. Depending on results of clinical response to induction therapy and restaging, patients were treated with surgery or radiotherapy. In total, 61 patients entered from March 2001 to April 2004. During 169 courses of induction chemotherapy, National Cancer Institute of Canada (NCI-C) grade III/IV leucocytopenia was reported in 35 courses (20.7%), NCI-C grade III/IV thrombocytopenia in 26 courses (15.4%) and NCI-C grade III/IV anaemia in six courses (3.6%). Main cause of cisplatin dose reduction was nephrotoxicity (12 courses). Most patients received three courses. There were no chemotherapy-related deaths. Three patients were not evaluable for clinical response. Twenty-eight patients had a partial response (48.3%, 95% CI: 36–61.1%), 24 stable disease and six progressive disease. After induction therapy, 30 patients underwent surgery; complete resection was achieved in 19 procedures (31.1%). Radical radiotherapy was delivered to 25 patients (41%). Six patients were considered unfit for further treatment. Median survival for all patients was 18 months. Response rate of accelerated cisplatin and high-dose epirubicin as induction chemotherapy for stage IIIa-N2 NSCLC patients is not different from more commonly used cisplatin-based regimen.

At present, treatment options for patients with stage IIIa-N2 non-small-cell lung cancer (NSCLC) are unclear. Whether surgery after induction therapy is of value for unselected patients with stage IIIa-N2 NSCLC is doubtful as in two recently presented large phase III trials no survival advantage for patients treated with definite chemoradiation as compared to the same treatment plus resection was found (Albain *et al*, 2005). In addition, no survival advantage was found for patients treated with surgery compared to thoracic radiation both after a response to induction chemotherapy (Van Meerbeeck *et al*, 2005).

In resectable stage IIIa-N2 NSCLC, the role of induction chemotherapy has been investigated in phase III randomised studies, comparing induction chemotherapy followed by surgery with surgery alone. In two small studies a survival benefit was found for the patients treated with induction chemotherapy (Roth *et al*, 1998; Rosell *et al*, 1999). Since then, a number of single arm phase II trials have been reported aiming to identify optimal chemotherapy and subgroups of patients that seem to benefit most of this treatment. For instance, a recent phase II trial reported upon the combination docetaxel–cisplatin as induction treatment followed by surgical resection. Mediastinal downstaging and complete resection proved to be very strong positive prognostic factors for survival, and 3-years survival of patients receiving neoadjuvant therapy plus complete resection was 70% (Betticher *et al*, 2003).

However, the optimal combination and dosage of induction chemotherapy is still unknown. Within past induction trials, cisplatin was thought to be an essential part of the combinations used but the other drugs necessary to improve response rate and survival were not identified. An alternative approach might be dose intensification of existing regimes. In NSCLC, the value of dose intensification is unknown. A feasibility and efficacy study of dose intensification of cisplatin and epirubicin in patients with relapsed NSCLC demonstrated that epirubicin can be escalated to 135 mg m^−2^ every 2 weeks provided that granulocyte-colony stimulating factor (G-CSF) support was added to prevent severe myelosuppression (Huisman *et al*, 2001). Response rate in this study was 33% (95% CI: 15–51%), which generated interest in evaluating this schedule as a first-line regimen for stage IIIa-N2 NSCLC and lead to this phase II trial. The schedule also is an attractive treatment option because it can be completed within a short time period. Owing to the frequently reported anaemia in the feasibility and efficacy study, erythropoietin was included in the trial as additional haematological support. In addition, the beneficial effects of erythropoietin on anaemia, functional status and quality of life of cancer patients receiving chemotherapy have been demonstrated by several large randomised (Abels, 1993; Cascinu *et al*, 1995; Savonije *et al*, 2005) and nonrandomised studies (Glaspy *et al*, 1997; Demetri *et al*, 1998). In this paper, the results from a multicenter phase II trial of accelerated cisplatin and high-dose epirubicin with G-CSF and erythropoietin support in stage IIIa-N2 NSCLC are reported.

## MATERIALS AND METHODS

### Patient criteria and study design

Between March 2001 and April 2004, patients with measurable stage IIIa-N2 NSCLC were entered in this prospective, nonrandomised phase II trial. Stage of disease had to be pathologically proven or confirmed in a multidisciplinary team of medical doctors. Methods to obtain pathological proof for mediastinal lymph node involvement were mediastinoscopy, thoracotomy, endoscopic ultrasound-guided fine-needle aspiration (EUS-FNA) or bronchoscopy with transbronchial needle aspiration (TBNA). Eligible patients were over 18 years old and had adequate haematological, renal and hepatic functions at the start of chemotherapy. Exclusion criteria were signs of cardiac failure, signs of active infection at the start of treatment, Eastern Cooperative Oncology Group (ECOG) performance status 3–4, other stage, and previous chemotherapeutic treatment. Ethics committees approved the trial at each participating center and written informed consent had to be obtained from all patients.

After pretreatment examination, patients received cisplatin 60 mg m^−2^ and epirubicin 135 mg m^−2^ both in 250 ml NaCl 0.9% on day 1 as an intravenous infusion for 30 min every 14-days for three consecutive courses. Haematopoietic growth factors were administered as erythropoietin subcutaneous (s.c.) at a dose of 30 000 IE one time per week and G-CSF s.c. at a dose of 300 *μ*g day^−1^ for patients with a body weight <60 kg and 480 *μ*g day^−1^ for patients with a body weight ⩾60 kg on days 3–12 of each course. To control emesis, ondansetron HCl and dexamethasone were administered intravenously two times per day in a dose of 8 mg and 20 mg, respectively. On days 2 and 3, patients received three times per day metoclopramide 20 mg rectal. Toxicity was measured according to the National Cancer Institute of Canada (NCI-C) grading system. Cisplatin dose was modified if creatinine clearance (using Cockroft-Gault formula) fell below 60 ml min^−1^, in case of significant hearing loss as demonstrated by audiogram, or neurotoxicity NCI-C grade II/III. Epirubicin dose was reduced to 120 mg m^−2^ when leucocytopenia and/or thrombocytopenia NCI-C grade IV lasting for more than 5 days occurred, in case of neutropenic fever and any other NCI-C grade III toxicity other than alopecia. The next chemotherapy course had to be postponed for 1 week with a maximum of 2 weeks in case of insufficient bone marrow recovery (leucocytes <3 × 10^9^ l^−1^ and/or platelets <100 × 10^9^ l^−1^). Follow-up investigations consisted of physical examination every 2 weeks; haemoglobin, white blood cell counts, differential counts and platelets on days 10 and 14 and liver/renal function before every course. Finally, chest röntgenograms (X-chest) were performed before every course, computer tomogram of the chest (CT-thorax) and fluoro-2-deoxy-D-glucose positron emission tomography (FDG-PET) before first course and after three cycles. CT-thorax and X-chest were used for measuring clinical response according to the response evaluation criteria in solid tumors (RECIST) (Therasse *et al*, 2000). At least two courses had to be completed for evaluation of clinical response, which was typically assessed after two of three courses. Complete response was defined as complete disappearance of all known tumour lesions. A partial response (PR) was defined as either (a) at least a 30% decrease in the sum of the longest diameter (LD) of target lesions taking as reference the baseline sum LDs or (b) complete disappearance of target lesions, with persistence (but not worsening) of one or more nontarget lesions. Progressive disease (PD): at least a 20% increase in the sum of LD of target lesions taking as reference the smallest sum LD recorded since the treatment started or the appearance of one or more new lesions and/or unequivocal progression of existing nontarget lesions. And finally, if there was neither sufficient shrinkage to qualify for PR nor sufficient increase to qualify for PD taking as references the smallest sum LD, it was defined as stable disease. This study was based on the Simon one sample two-stage testing procedure (Simon, 1989). A response rate of 75% or more warranted further investigation of the chemotherapy regimens used, and a response rate of 55% or less warranted rejection. The upper limit of 75% was chosen because it is close to the observed response rate of the gemcitabine–cisplatin combination which is the most widely used induction regimen on the European continent (Van Zandwijk *et al*, 2000). In addition, since it is intensified chemotherapy that is applied with potentially more severe side effects we choose to set the upper limit on 75% for the power calculation of the study.

### Restaging and postinduction treatment

Restaging had to be performed to decide whether surgery or radiotherapy was the best therapeutic option after induction chemotherapy. Methods used for restaging were CT-thorax, FDG-PET, X-chest, mediastinoscopy and EUS-FNA.

Patients without persisting N2-disease and who were medically fit to proceed to surgery underwent thoracotomy, with the intention to achieve a complete resection, preferably by lobectomy or else pneumonectomy. Mediastinal lymph node dissection was performed with each resection. Patients with incomplete or no resection at thoracotomy were offered postoperative radiotherapy. The patients with persisting N2-disease and/or PD at restaging were treated with radical thoracic radiotherapy, typically consisting of 46 Gy in 23 fractions delivered to the primary field and 13.8 Gy in 23 fractions delivered as a boost on the tumour. This total of 59.8 Gy was applied in a 5 days week^−1^ scheme with duration of 5 weeks. Lower dosages were delivered as palliative radiotherapy.

### Statistical analysis

Time to postinduction treatment is dated from first day of last course of induction chemotherapy to the date of thoracotomy or first day of radiotherapy.

Survival is calculated from day 1 of the first course (start treatment) until death or 1 May 2005. The survival curve is estimated by the Kaplan–Meier method (Kaplan and Meier, 1958). Disease-free survival is defined as the period from start treatment to the date of disease progression, relapse or death.

## RESULTS

### Patient characteristics

In total, 62 consenting patients were entered from March 2001 to April 2004 in four hospitals in The Netherlands. One patient withdrew informed consent before the start of study treatment. In Table 1, the characteristics of all remaining 61 patients are summarised. The study group consisted of 44 men and 17 women with a median age of 63 years (range 40–79) and median ECOG performance status 1. Largest histological subgroup was squamous cell carcinoma. In all, 55 patients had pathologically proven stage IIIa-N2 NSCLC, by mediastinoscopy (33), EUS-FNA (16), thoracotomy (4) and bronchoscopy with TBNA (2). Six patients were assigned to stage IIIa-N2 on the basis of FDG-PET, CT-thorax and/or expertise of a multidisciplinary team of lung cancer specialists.

### Toxicity

All patients who started protocol treatment are evaluable for toxicity. In total 169 courses of chemotherapy were administered, with a median of three (range 1–4). Median relative dose intensity (RDI) of cisplatin was 99.7% (range 49.7–105.3) and of epirubicin 100% (range 59.3–106.5). A dose reduction of cisplatin was performed in 15 courses because of low creatinine clearance (12), ototoxicity NCI-C grade II (1) and deterioriation of performance status (2). Two of these 15 courses contained no cisplatin at all. Epirubicin dose has been reduced in two courses due to deterioriation of performance status. NCI-C grade III/IV leucocytopenia was reported in 35 courses (20.7%), NCI-C grade III/IV thrombocytopenia in 26 courses (15.4%) and NCI-C grade III/IV anaemia in six courses (3.6%). Neutropenic fever followed by hospitalisation occurred in one patient and anaemia was corrected by red blood cell transfusion in two patients. Nausea, vomiting and alopecia were experienced frequently, but never higher than NCI-C grade II/III.

### Clinical response and restaging

Three patients were not evaluable because of interruption of protocol treatment after one course due to leucocytopenia NCI-C grade III/IV (2) and sepsis as a result of pneumonia (1), leaving 58 patients assessable for clinical response. There were no complete remissions, a PR was observed in 28 patients, 24 had stable disease and six patients progressed. The objective response rate (ORR) was therefore 48.3% (95% CI: 36.0–61.1%).

Restaging was performed in evaluable patients without PD at clinical response evaluation (*n*=52). Owing to logistical reasons in only 46 patients a restaging FDG-PET was performed. Twelve patients underwent a mediastinoscopy, one of them also underwent an EUS-FNA along with seven other patients. N2-positive lymph nodes were detected in four (mediastinoscopy) and three (EUS-FNA) procedures. Seventeen out of 19 patients who underwent mediastinoscopy and/or EUS-FNA for restaging were also evaluated by FDG-PET. Compared to mediastinoscopy and/or EUS-FNA, FDG-PET predicted correct nodal status in 13 patients (76.4%), was false-positive in two (11.8%) and false-negative in two additional patients (11.8%).

### Postinduction treatment

Median time to postinduction treatment was 51 days (range 10–142), for surgery 49 days (range 10–142) and for radiotherapy 55 days (range 16–112). Table 2 summarises the postinduction treatment characteristics. In total 30 patients were approved for surgery, including one patient who progressed under cisplatin/epirubicin but had stable disease after three additional courses of taxotere/gemcitabine. Six patients were surgically explored but had no resection because of positive mediastinal lymph nodes confirmed during thoracotomy (3) or progression to stage IIIb/IV (3). Surgery with resection was performed in 24 patients, 10 patients had a pneumonectomy, 13 a lobectomy and one a wedge resection.

Frequently encountered complications of surgery were anaemia, atrial fibrillation and infectious complications (*n*=9) of which two had empyema. The latter patients fully recovered after treatment with appropriate antibiotic therapy. Complete resection was achieved in 19 patients, five resections were defined incomplete because of microscopic residual disease. Pathological examination of the resected specimens showed complete pathological response in one patient and N2-disease in nine patients. Of those nine patients, three had a negative mediastinoscopy and two a negative EUS-FNA at restaging. Radical radiotherapy was administered after resection to three patients. Patients who underwent thoracotomy without resection were afterwards treated with radical (2) or palliative (4) radiotherapy. After being restaged negative for surgery, 25 patients proceeded to thoracic radiotherapy (10 radical and 15 palliative). Six patients did not have any postinduction treatment due to deterioriation of performance status.

### Survival and disease-free survival time

**Figure 1:**
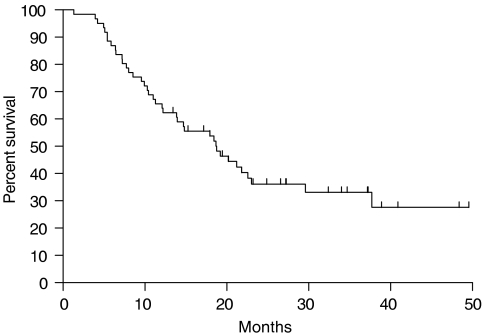
Overall survival (*n*=61).

Reference date for survival analysis was set at 1 May 2005 and at that date 39 patients have died (63.9%). They died without evidence of disease (5), with disease (26), from other cause (5) or from unknown cause (3). Results of survival estimation are shown in Figure 1. After a median follow-up period of 17 months, the median duration of survival for all patients was 18 months (range 1–50+), for resected patients 27 months (range 6–50) and for patients treated with radiotherapy (including explored but unresected patients) 14 months (range 1–34). In all, 34 patients (55.7%) have developed a relapse of lung cancer, either in the lung (17), brain (9), bone (3) or other sites (5). No evidence of disease was reported for 17 patients (27.9%). Median disease-free survival time was 11 months (range 3–28).

## DISCUSSION

Although every chemotherapy regimen becomes more toxic in higher dose, epirubicin at a dose of 135 mg m^−2^ can be given without major side effects, provided G-CSF support is offered (Huisman *et al*, 2001). In this trial leucocytopenia NCI-C grade III/IV was observed in 20.7% of the courses but only one patient developed neutropenic fever. Erythropoietin administration has confirmed its preventive role during chemotherapy, since only 3.6% of all courses were associated with NCI-C grade III/IV anaemia, in two of these patients followed by red blood cell transfusion. No patients died during chemotherapy, and both cisplatin and epirubicin were given with a median RDI of almost 100%.

The results from clinical response, 28 PRs out of 58 evaluable patients (ORR 48.3%, 95% CI: 36.0–61.1%), are in the same range as comparable neoadjuvant cisplatin-based phase II trials: In a study of 90 patients (stage IIIa N2 NSCLC) with docetaxel and cisplatin, ORR was 66% (95% CI: 55–75%) (Betticher *et al*, 2003). The Toronto trial (65 patients) with mitomycin, vindesine and cisplatin for stage IIIa unresectable NSCLC reported an ORR of 67.7% (Burkes *et al*, 2005) and a trial (47 patients) of gemcitabine and cisplatin for biopsy-proven stage IIIa-N2 NSCLC reported an ORR of 70.2% (95% CI: 55.1–82.7%) (Van Zandwijk *et al*, 2000). Finally, a trial (42 patients) of gemcitabine–cisplatin–paclitaxel for stage IIIa(N2)/IIIb inoperable NSCLC reported an ORR of 71% (95% CI: 57.2–84.7%) (Cappuzzo *et al*, 2003). In particular, an analysis of the EORTC 08941 phase III study which used a variety of induction chemotherapies in stage IIIa-N2 NSCLC reported an ORR of 59% (95% CI: 54–64%) for cisplatin containing regimens (Van Meerbeeck *et al*, 2003). Accurate response assessment after induction treatment is crucial, since only those patients with mediastinal downstaging will benefit most of surgery. CT-thorax often underestimates pathological response (Lee *et al*, 2000; Margaritora *et al*, 2001). Another option is FDG-PET, which role in NSCLC is not fully understood. Hoekstra *et al* (2005) reported that after one course of induction chemotherapy for patients with stage pIIIa-N2 NSCLC, responders and nonresponders can be separated by FDG-PET. However, assessment of pathological response in the mediastinal lymph nodes is not reliable. Two small studies compared surgical staging with FDG-PET and reported that after induction therapy, a correct nodal status was predicted by FDG-PET in 48–52% (Akhurst *et al*, 2002; Port *et al*, 2004). In this trial, 19 patients underwent restaging with mediastinoscopy and/or EUS-FNA and 17 of these had also been evaluated by FDG-PET. Prediction of nodal status was correct in 13 patients (76.4%), overstaged in two (11.8%) and understaged in two patients (11.8%). The role of FDG-PET as predictor of pathological response after induction therapy for NSCLC is unclear and has to be investigated in trials with larger patient numbers.

The most important prognostic factor for survival is the presence of N2-disease after induction therapy. Eradicated nodal status is related to higher survival rates (De Leyn *et al*, 1999; Bueno *et al*, 2000; Sawabata *et al*, 2003). At present, N2-disease can only be accurately detected by pathological response evaluation. Mediastinoscopy is a good option for this purpose, but more techniques are available, especially EUS-FNA. A recent study reported that the combination of mediastinoscopy and EUS-FNA detected more patients with N2-disease than mediastinoscopy alone (Annema *et al*, 2005). In our study, seven patients with negative mediastinoscopy had a resection and pathological examination of the specimens showed N2-disease in three (42.9%) patients. Endoscopic ultrasound-guided fine-needle aspiration was negative for five patients, they all had a resection, and their pathological examination showed N2-disease in two (40%) patients. Endoscopic ultrasound-guided fine-needle aspiration is an useful method for pathological staging in NSCLC and its role has to be defined for response evaluation.

Median time to postinduction treatment was 51 days (range 10–142). It is likely that this would have implications for survival due to accelerated repopulation after chemotherapy (Kim and Tannock, 2005). Compared to the EORTC 08941 study (Van Meerbeeck *et al*, 2005), the median time to postinduction treatment is identical (51 days, range 17–113).

Median survival for all 61 patients in this trial is 18 months (range 1–50+) and 27 months for resected patients, this is similar to the studies mentioned before.

In conclusion, response rate of accelerated cisplatin and high-dose epirubicin as induction chemotherapy for stage IIIa-N2 NSCLC patients is not different from more commonly used cisplatin-based regimen. Based on the statistical design of this study that rejects further exploration at a response rate of 55% or less, investigating this chemotherapy combination in phase III trials is not recommended.

## Figures and Tables

**Table 1:** Patient characteristics (*n*=61)

**Characteristic**	**No. of patients**	**%**
*Sex*		
Male	44	72.1
Female	17	27.9
		
*Age (years)*		
Median	63	
Range	(40–79)	
		
*ECOG performance status*		
0	24	39.3
1	37	60.7
		
*Histology*		
Squamous	29	47.5
Adenocarcinoma	18	29.5
Adenosquamous	1	1.6
Large cell	13	21.3
		
*Clinical stage*		
Tx N2 M0	2	3.3
T1 N2 M0	12	19.7
T2 N2 M0	34	55.7
T3 N2 M0	13	21.3
		
*Biopsy-proven N2-disease*		
Positive	55	90.2
Not performed	6	9.8
		
*Clinical response*		
Partial response	28	48.3
Stable disease	24	41.4
Progressive disease	6	10.3
Not evaluable	3	—
		
*Postinduction treatment*		
None	6	9.8
Surgery (resected)	24	39.3
Surgery (explored, no resection)	6	9.8
Radiotherapy	25	41.0

ECOG=Eastern Cooperative Oncology Group.

**Table 2:** Postinduction treatment characteristics (*n*=61)

**Characteristic**	**No. of patients**	**%**
*Surgical procedure*		
Pneumonectomy	10	16.4
Lobectomy	13	21.3
Wedge resection	1	1.6
Exploration	6	9.8
		
*Completeness of resection*		
R0		19
R1		5
Unresectable	6	9.8
		
*Pathological staging*		
Complete response	1	1.6
T1-3 N0 M0	10	16.4
T1-3 N1 M0	4	6.6
T1-3 N2 M0	9	14.8
No specimens	6	9.8
		
*Radiotherapy*		
After restaging		
Radical	10	16.4
Palliative	15	24.6
		
After surgery		
Radical	5	8.2
Palliative	4	6.6
No postinduction treatment	6	9.8
